# Querying Recombination Junctions of Replication-Competent Adeno-Associated Viruses in Gene Therapy Vector Preparations with Single Molecule, Real-Time Sequencing

**DOI:** 10.3390/v15061228

**Published:** 2023-05-24

**Authors:** Mitchell Yip, Jing Chen, Yan Zhi, Ngoc Tam Tran, Suk Namkung, Eric Pastor, Guangping Gao, Phillip W. L. Tai

**Affiliations:** 1 Horae Gene Therapy Center, UMass Chan Medical School, Worcester, MA 01605, USA; 2 Spirovant Sciences, Inc., Philadelphia, PA 19104, USA; 3 Department of Microbiology and Physiological Systems, UMass Chan Medical School, Worcester, MA 01605, USA; 4 Li Weibo Institute of Rare Diseases Research, UMass Chan Medical School, Worcester, MA 01605, USA

**Keywords:** adeno-associated virus, replication-competent AAV, single molecule, real-time sequencing, AAV-genome population sequencing

## Abstract

Clinical-grade preparations of adeno-associated virus (AAV) vectors used for gene therapy typically undergo a series of diagnostics to determine titer, purity, homogeneity, and the presence of DNA contaminants. One type of contaminant that remains poorly investigated is replication-competent (rc)AAVs. rcAAVs form through recombination of DNA originating from production materials, yielding intact, replicative, and potentially infectious virus-like virions. They can be detected through the serial passaging of lysates from cells transduced by AAV vectors in the presence of wildtype adenovirus. Cellular lysates from the last passage are subjected to qPCR to detect the presence of the *rep* gene. Unfortunately, the method cannot be used to query the diversity of recombination events, nor can qPCR provide insights into how rcAAVs arise. Thus, the formation of rcAAVs through errant recombination events between ITR-flanked gene of interest (GOI) constructs and expression constructs carrying the *rep*-*cap* genes is poorly described. We have used single molecule, real-time sequencing (SMRT) to analyze virus-like genomes expanded from rcAAV-positive vector preparations. We present evidence that sequence-independent and non-homologous recombination between the ITR-bearing transgene and the *rep*/*cap* plasmid occurs under several events and rcAAVs spawn from diverse clones.

## 1. Introduction

Several gene therapy treatments based on adeno-associated virus (AAV) vectors have now gained approval for commercial use in multiple countries [1,2,3]. Despite the upward trajectory for developing AAV-based gene therapies, manufacturing challenges still present bottlenecks for the field. Following the production of AAV vectors, preparations typically undergo a series of diagnostic assays to determine titers, purity, the abundance of full versus empty capsids, and the presence of DNA contaminants packaged into virions [4]. These DNA contaminants can originate from components used during the production process, which are typically plasmid fragments and segments of host-cell genomic DNA [4]. These contaminants are thought to be innocuous, especially if they exist at less than 10 ng of residual cellular DNA and less than 100 ng for residual plasmid DNA per 1E12 vg of purified AAV vector [4,5]. However, much of this belief results from a lack of evidence linking adverse effects to impurities in preparations. Nevertheless, two forms of contaminants that are clear concerns, even at low relative abundances, are adventitious viruses and replication-competent (rc)AAV.

**Figure 1:**
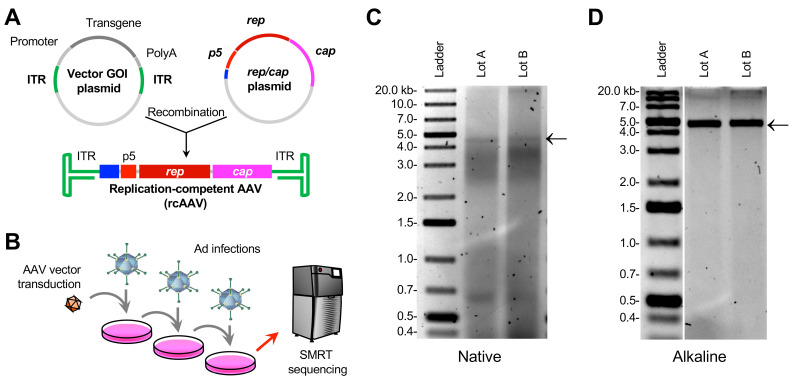
Cell-based amplification of rcAAVs present in vector preparations reveals ~4.7-kb genomes. (**A**) Illustration of the formation of rcAAVs by recombination between the ITR-bearing GOI vector and the *rep*/*cap* expression plasmid. Units that comprise the rcAAV: ITRs (green), the Ad ITR (blue), *p5* promoter (light red), *rep* (dark red), and *cap* (magenta) are shown. (**B**) Schematic of rcAAV amplification and characterization. AAV vectors were used to transduce HEK293 cells in the presence of Ad helper virus. Crude lysates were collected, subjected to a freeze/thaw cycle, clarified, and used to infect HEK293s. This was done two more times. Crude lysates from the third round of infection was subjected to DNA purification and SMRT sequencing analyses. (**C**,**D**) Analysis of crude lysates by agarose gel electrophoresis. Native (**C**) and alkaline denaturing (**D**) gel electrophoresis of Lots A and B. The 4.7-kb bands (black arrows) represent AAV genomes purified from crude lysate.

Adventitious viruses, which are natural viruses that originate from serum, cell lines, or other environmental sources, are inherently problematic. They may elicit strong immunological responses in patients or can be pathogenic. Adventitious viruses can typically be detected by PCR using primers/probes against known viral sequences in plasmid or vector preparations, or they can be detected by cytopathic effect (CPE) assays [6]. In contrast, rcAAVs are formed from the recombination of ITR-bearing DNAs with *rep* and *cap* sequences (Figure 1A) [7,8,9]. Adverse effects of rcAAVs are unknown, but they are presumed to be non-pathogenic like their natural counterparts. Similar to wildtype AAVs, rcAAVs are presumed to be replication-deficient without a helper virus, such as adenovirus, herpesvirus, and papillomaviruses [10]. Since most production platforms lack the use of infectious helper viruses, rcAAVs that are formed are not expanded. Therefore, their outcomes following administration into patients remain unexplored. Clinical AAV vectors are based on serotypes that are highly tropic to the liver, which are natural harbors for adenoviruses and herpesviruses [11,12]. Since rcAAVs can theoretically reside as stable episomes along with therapeutic vectors, they can potentially be rescued upon natural infection by any helper virus common in the human population. Therefore, the potential for rcAAVs to replicate in the host following the administration of an AAV-based gene therapeutic is real. This is a particular concern when non-natural, engineered capsids that harbor alternative functions or confer new tropism profiles are used in gene therapy. Many of these novel capsids are not tested in the context of a replicating virus. In contrast to other forms of contaminants, rcAAVs are difficult to detect [13], as they share sequences with packaging components. They are also not detectable in CPE assays, since they are not cytopathic on their own [14]. To detect rcAAVs, vector preparations are tested in cell culture. Clarified lysates from transduced cells are passaged several times in the presence of adenovirus and then the crude lysates are subjected to qPCR to detect *rep*. Sanger sequencing can also be performed to query the recombination junctions at the 5′ end of the *rep* gene and 3′ end of the *cap* gene with ITRs [9]. Although these approaches are standard for detecting rcAAVs, these methods cannot be used to profile the diversity of rcAAVs arising from stochastic recombination events and therefore limits our capacity to develop strategies to eliminate the production of rcAAVs through platform improvements.

Next-generation sequencing (NGS), specifically long-read sequencing approaches that can capture reads longer than 5 kb in length, have helped to reveal the genome compositions packaged into AAV vectors [15,16,17]. The use of single molecule, real-time (SMRT) sequencing and AAV genome population sequencing (AAV-GPseq), which can capture vector DNAs as an intact read, have shown that chimeric genomes can be generated through recombination [15,16]. In fact, nearly 50% of contaminating genomes have been demonstrated to harbor ITRs [15,16], suggesting that these species are actively packaged into assembled capsids and can be replicated via the ITRs. The capacity for AAV-GPseq to characterize ITR-bearing contaminants raised the question whether it can be used to profile rcAAVs.

In this study, we have used SMRT sequencing to analyze rcAAVs amplified from an AAV vector preparation. We present evidence that recombination between *rep*/*cap* plasmids and transgene cassette plasmids can form due to several events and is not sequence-dependent. These findings suggest that random recombination, which is prevalent during the packaging of AAV vectors [15], can produce rcAAVs as long as all the components for replication and packaging are present. Importantly, this new knowledge has helped to improve our knowledge on how rcAAVs form.

## 2. Materials and Methods

### 2.1. Plasmid and Vector Production

The AAV vectors described in this study were produced by plasmid transient transfection of HEK293 cells (ThermoFisher), grown in serum-free medium in bioreactors, using the FectoVIR™ platform following manufacturer’s recommended procedures (https://www.polyplus-transfection.com/) (accessed on 9 September 2020). After production, cells were lysed and AAV-GOI vectors were purified using two-column chromatography [18].

### 2.2. Propagation of rcAAV

HEK293 cells (ATCC, CRL-1573) that were seeded in T75 flasks were co-infected with the AAV-GOI vectors and wildtype adenovirus serotype 5 (Ad5). After three days of culturing, cell lysates were subjected to three freeze–thaw cycles, followed by Ad5 heat-inactivation. Aliquots of collected and clarified supernatant were used in the second round of amplification in freshly prepared HEK293 cells in the presence of Ad5. A third round of amplification was performed with samples prepared after a second amplification round following the same procedure. Clarified cell lysate samples were collected after the third amplification round and analyzed using qPCR for the presence of *rep2* DNA.

### 2.3. Vector DNA Extraction and Agarose Gel Electrophoresis

Third-passage crude lysates from two vector lot preparations, referred herein as Lots A and B, were purified using phenol:chloroform:isoamyl alcohol (25:24:1) and ethanol precipitation as described previously [15]. Samples underwent heating and strand annealing in an annealing buffer (25 mM NaCl, 10 mM Tris-HCL [pH8.5], 0.5 mM EDTA [pH 8.0]) at 95 °C for 5 min and cooled to 25 °C (1 min for every −1 °C) on a thermocycler (Eppendorf Mastercycler). Purified vector DNAs were then subjected to standard agarose and denaturing alkaline gel electrophoresis. Gels were stained with ethidium bromide to visualize the rcAAV-containing bulk DNA isolated from lysates [19].

### 2.4. SMRT Sequencing and AAV-GPseq

Purified vector DNAs were subjected to singe molecule, real-time (SMRT) sequencing using the UMass Chan Medical School Deep Sequencing Core. Libraries for vector DNAs were constructed using the Express Template Prep Kit 2.0 (End Repair/A-tailing) (PN 100-938-900) and ligated to indexed SMRTbell adapters with the Barcoded Overhang Adapter Kit (PN 101-628-400/500). Libraries were pooled and purified using 1.8× AMPure beads. Sequencing was performed on a Sequel II instrument. Vector Lots A and B were sequenced on individual flow cells.

### 2.5. Bioinformatics

Subreads from each library for Lots A and B underwent preprocessing steps prior to analysis as described previously [17]. Briefly, subreads were first processed through recalladapters 9.0.0 with the following parameters: -minSnr = 2.0 and -disableAdapter Correction. This step recovered long palindromic sequences that were artificially cleaved from subreads generated by SMRT-Link 10. Subsequent subreads were then processed using the circular consensus sequencing (CCS) tool in SMRT Link version 10.0.119588 with the following parameters: -minSnr = 3.75, -min = passes = 2 and -bystrand.

The following bioinformatic workflows were conducted using the Galaxy platform [20]. Resulting consensus fastq files were mapped to the various references reported in the study using the Burrows–Wheeler aligner-maximal exact match (BWA-MEM) [21]. Aligned reads were visualized with the Integrated Genomics Viewer (IGV) tool version 2.14.0 with soft-clipping on. Recombination junctions were identified using the cutadapt tool to isolate either the 5′ or 3′ junctions using unique sequences. Junction forms were defined with custom scripts using USEARCH and clusterfast. Reads were clustered with 95% similarity using USEARCH v10.0.240 with the command -cluster_fast and parameter -id 0.95. Clusters with more than one member and with distinct junctions were selected.

## 3. Results

### 3.1. Amplification of rcAAVs

For this investigation, we selected to analyze clarified cell lysates that were positive for rcAAVs and originating from an AAV vector preparation consisting of a ~4.9-kb single strand genome. The root construct, has a ~4.2-kb cDNA driven by a short synthetic promoter [22], hereinafter referred to as the “gene of interest” (GOI). The single-strand vector construct was packaged with a non-natural chimeric capsid [23] using the triple plasmid transfection method and purified by two-column chromatography [18]. Potential rcAAVs present in vector lots were amplified in HEK293 cells in three rounds (Figure 1B). Clarified cell lysate samples collected after the third amplification round were analyzed using qPCR for the presence of *rep2* DNA. Two lots, reported here as Lot A and Lot B, were found to be positive for rcAAV.

### 3.2. Isolation of DNA from Crude Lysates of Cells Transduced with Expanded rcAAVs Yields Heterogeneous ~5-kb Genomes

Third-round crude lysates from Lots A and B were directly processed for DNA extraction. Native agarose gels of the bulk DNA material showed that the DNA of both lots was highly heterogeneous (Figure 1C). Notably, a faint band was observed between the 4-kb and 5-kb markers. When the DNA was subjected to alkaline gel electrophoresis, a prominent band near the 5-kb marker was observed (Figure 1D). The contrasting gels suggested that the main sizes observed are those that are near 5 kb in size, but are highly heterogeneous, since native gels showed smears (Figure 1C).

We next aimed to sequence the genomic material that was isolated. Since we did not wish to introduce any additional bias for genome representation, we opted to directly use the total genomic material for library preparation and SMRT sequencing. No size selection, target-specific enrichment, or amplification of *rep*/*cap* sequences was therefore applied to the crude lysate preparation. In contrast to conventional means of isolating vector genomes for AAV-GPseq [17], the preparation method used here did not differentiate between genomes that were packaged into capsids (i.e., DNase resistant) versus those that were not. Material from third-round lysates of each lot was ran on separate SMRT cells to maximize the representation for each lot. Lot A yielded 1.8 million reads, while Lot B produced 6.5 million reads (Table 1). Since the SMRT libraries were generated without any selection criteria, we expected that the majority of the reads would be related to the host cell genome. When reads were aligned to the human genome (hg38), we observed 1.4 million (76.98%) and 5.38 million (82.98%) mapped reads for Lots A and B, respectively. We next aligned the reads to the *rep*/*cap* plasmid and found that Lot A had 230,787 (12.55%) mapped reads, while Lot B yielded 217,501 (3.35%) mapped reads. Interestingly, reads aligning to the AAV-GOI reference were at a much lower percentage, 103,613 (5.63%) and 31,127 (0.49%) for Lots A and B, respectively (Table 1). This finding suggested that by the third round of lysate passaging, reads containing *rep*/*cap* sequences occurred in a higher percentage of captured DNAs than those related to the GOI.

### 3.3. Transgene-Related Sequences Were Rare in rcAAV-Enriched Lysates

**Figure 2:**
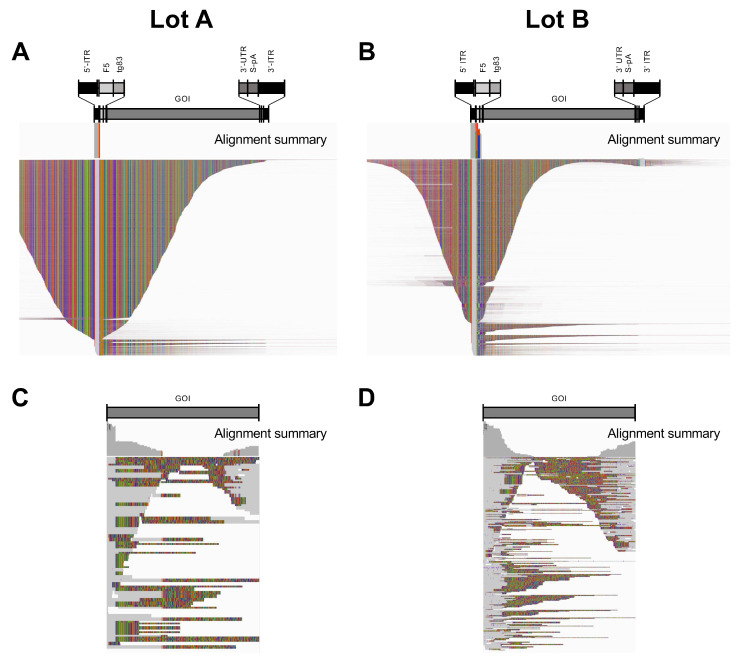
Reads from Lots A and B aligned to the AAV-GOI transgene reference. (**A**,**B**) IGV displays of reads from Lots A and B align only to the AAV2 ITRs of the ~4.3-kb GOI reference. (**C**,**D**) Alignments to the GOI reference alone. Regions of read matches (gray), mismatches (colored), and deletions/insertions (speckles) are shown in squished display.

The relatively high number of aligned reads to the AAV-GOI reference suggested that many of the non-host cell genomes were attributed to the GOI transgene (Table 1). However, when these aligned reads were visualized, the vast majority only mapped to the ITR (Figure 2A,B). This finding demonstrated that serially amplified rcAAVs contain ITRs, but do not harbor transgene genomes. To verify this observation, we aligned reads to the open reading frame (ORF) of the GOI alone (Figure 2C,D). Reads mapping to this reference accounted for 155 (0.84%) and 494 (0.76%) reads for Lots A and B, respectively. Importantly, none of the reads spanned the entire ORF (Figure 2C,D), indicating a lack of detectable full-length vector genomes amplifying along with rcAAVs.

### 3.4. Mapping Reads to a Reconstructed rcAAV Reference Revealed ITRs That Were Contiguous with rep and cap Sequences

**Figure 3:**
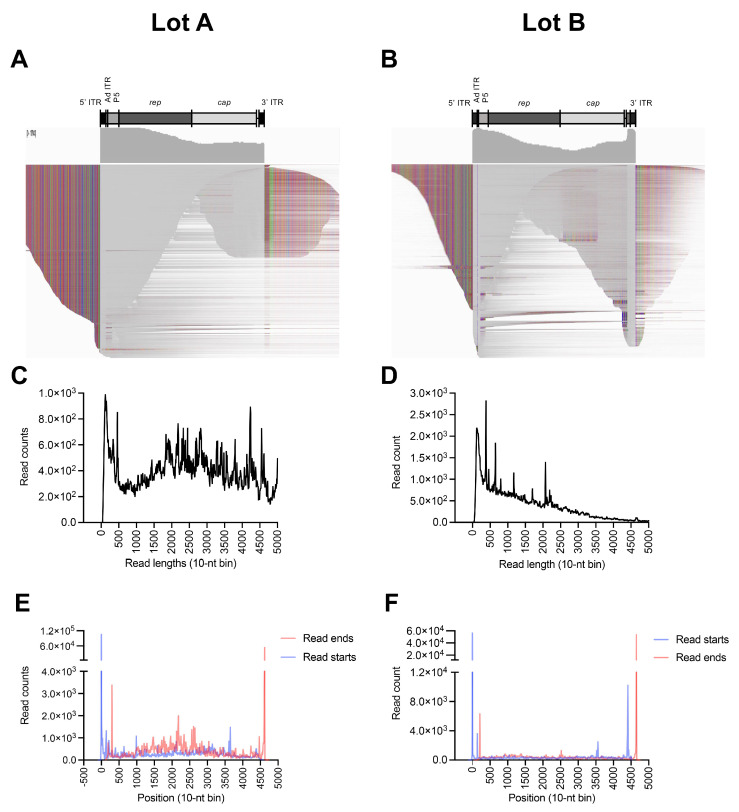
Reads from Lots A and B aligned to modified *rep*/*cap* references. (**A**) IGV displays of reads from Lot A showing rcAAV with an Ad-ITR following the 5′ ITR. (**B**) IGV display of reads from Lot B showing rcAAV with a partial *p5* promoter following the 5′ ITR and an additional *p5* promoter insert preceding the 3′ ITR. Regions of read matches (gray), mismatches (colored), and deletions/insertions (speckles) are shown in squished display. (**C**,**D**) Lengths of aligned reads. (**E**,**F**) Histogram showing the aggregation of read alignment starts (blue) and ends (red).

We next aimed to understand the genome structures of the rcAAVs. We extracted a handful of reads that covered the 5′ end of the *rep* ORF and 3′ end of the *cap* ORF. Using these sample reads, we constructed a reference containing the recombination junctions with flip-configured ITRs. The SMRT reads from Lots A and B were then aligned to the rcAAV reference (Figure 3A,B). As predicted, the majority of reads that mapped to the ITRs showed contiguous mapping to the *rep* or *cap* ORF sequences, confirming that rcAAV genomes carry ITRs that have recombined with the *rep*/*cap* sequences. Interestingly, reads spanning the *cap* ORF retained the original chimeric capsid sequence. This result lends evidence that serial passaging in cell culture did not cause evolution of the rcAAV genomes after three rounds. We also observed that the majority of these reads do not span from ITR to ITR, but only span either the 5′ or 3′ ITRs as unresolved genomes (Figure 3A,B). Since the genomes were isolated from whole-lysates, it is likely that the species captured was undergoing replication and/or represent pre-resolved genomes. Additionally, the represented reads are self-complementary genomes with configurations containing the unresolved ITR in the middle of the read. This representation likely reflects the preferential ligation of the SMRTbell adaptor to double-strand free ends that result from the cleavage of the single-stranded DNAs away from the double-strand region of the genome as described previously [17]. Interestingly, Lot A showed a broader distribution of reads with lengths of 4–5 kb (Figure 3C), while Lot B had more shorter reads (Figure 3D). Of note, SMRT sequencing is known to favor the representation of shorter DNA species; thus, the abundances observed for rcAAV reads may reflect this bias.

To demonstrate the abundance of reads that capture the full ITR, the accumulation of the start and end positions of the aligned reads were plotted (Figure 3E,F). The majority of reads align at the termini of the references, demonstrating that the rcAAV genomes contain full ITRs.

**Figure 4:**
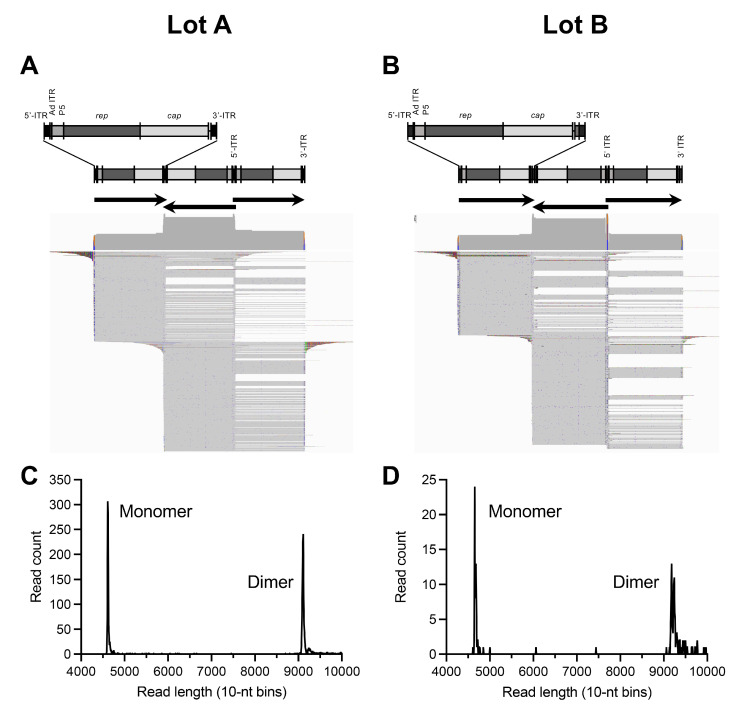
Reads aligned to unresolved trimer references show dimer genomes. (**A**,**B**) Reads from Lots A and B were mapped to their respective trimer references which reveal dimer genomes containing unresolved ITRs. Trimer references consist of three copies of the vector reference with arrows indicating the transgene direction. (**C**,**D**) Abundance of monomer and dimer species as revealed by read lengths distributions.

Wild-type AAV genomes are replicated by rolling-hairpin replication and require the genomes to be flanked by ITRs at both ends [24]. Since the majority of reads only covered one ITR, we aimed to profile genomes that spanned from ITR to ITR to ensure that the species under analysis are true replicative forms. Some of the reads were also predicted to persist as non-resolved dimers, since many of the genomes detected were unresolved (Figure 3A,B). We therefore selected reads that aligned to the reference from ITR to ITR and realigned them to an unresolved trimer (Figure 4). Many of the genomes were in fact found to be dimers (2× units), existing at 44% (Lot A) and 35% (Lot B) of all ITR-to-ITR-spanning reads (Figure 4C,D). Among the unresolved genomes, we did not observe a high degree of mutated ITRs, suggesting that these captured genomes are simply unresolved.

### 3.5. Formation of rcAAVs Occur via Non-Specific Recombination Events at the 5′ and 3′ Ends of the rep/cap Genes

We next aimed to determine whether recombination occurred at distinct sequences. We selected reads that span from ITR to ITR and isolated sequences that were directly adjacent to the ITRs to profile the diversity of junctions at the 5′ and 3′ ends. Among Lot A reads, we discovered seven junction types at the 5′ end that appeared more than once (Table 2). The majority of the junctions (designated A-5.1 to A-5.6) contained Ad-ITR, which was present within the original *rep*/*cap* plasmid construct profiled in this study. This finding suggested that the Ad-ITR is the driver for recombination, as described in a previous study [8]. The A-5.1 form was revealed to be the dominant species, making up 97.65% of all junction types. All other junctions had under 1% representation. Interestingly, five reads (0.16%, designated A-5.7) had a junction that lacked Ad-ITR. All of the junctions contained nearly intact ITRs with only one or four nucleotides missing from the 3′ end of the ITR. The A-5.2 form retained 15 nucleotides downstream of the ITR, reading into transgene plasmid. Three forms (A-5.4, A-5.5, and A-5.6) have 3–5 nt insertions of unknown origin within the Ad-ITR. For Lot B, we observed eleven forms of recombination junctions (Table 2). Interestingly, only two forms contained Ad-ITR sequences (B-5.6 and B-5.7), and these were represented at less than 1% of the reads. The two most dominant forms (B-5.1, 47.79%; and B-5.2, 38.76%) lack Ad-ITR sequences. These findings suggest that Ad-ITRs do not drive recombination or rcAAV formation.

Examination of the 3′ end of full-length rcAAVs revealed less diversity at the junctions (Table 3). Lot A only showed one form, consisting of the 3′ end of the *cap* gene recombined with the full ITR. Lot B showed three forms, all containing partial *p5* promoter sequences. Form B-3.1 was represented in 89.51% of the reads, while form B-3.2 was represented in 8.13% of the reads. These data support the notion that the Ad-ITR is not required for recombination and formation of rcAAVs, as the 3′ end of the *cap* gene lacks an Ad-ITR in the transgene cassette design. Additionally, the data suggest that recombination may have occurred at the 3′ end first and then recombination at the 5′ end may occur afterwards, spawning a greater diversity of recombination junctions at the 5′ end of rcAAVs. However, since we have only examined two AAV lots with detectable rcAAVs, the higher diversity at the 5′ ends may be coincidental. Nevertheless, our results show that recombination to form rcAAVs is achieved via non-homologous recombination and is not promoted by any clear and obvious sequence motif within the AAV-GOI or *rep*/*cap* plasmid.

## 4. Discussion

AAV-based gene therapy has made significant strides in the recent years. Three treatments have gained commercial approval in 2022 [2,3] and more are in development. Unfortunately, there is no standard way to produce AAV vectors, and quality control definitions are consistently being revised. To date, the presence of rcAAVs have not been a major concern for the gene therapy field. However, given new knowledge on the stable integration of AAV vector genomes into host chromosomes [25,26,27], the presence of rcAAVs may be more of a safety concern than previously acknowledged. One striking finding from our investigation is that the rcAAV reported in this study harbored the non-natural chimeric capsid, which propagated in cell culture while retaining the original capsid sequence. Although AAVs are considered non-pathogenic with low immunogenicity profiles, the finding that rcAAVs with non-natural capsids can be expanded may potentially pose a risk. Many clinical vectors now under investigation use capsids that are engineered to have unique tropisms as the natural serotypes are less than ideal for many disease targets. However, they have yet to be tested for potential pathogenicity in the context of a replication-competent virus. The advancement of new engineered viruses that are tropic to tissues and cell types beyond the capability of wildtype AAVs, can evade immune recognition, or harbor the ability to efficiently traverse the blood brain barrier may raise concerns if the target organs also harbor helper viruses.

It was previously suggested that the Ad-ITR (for plasmid designs that contain them) and ten nucleotides of the D sequence (5′-AGGGGTTCCT-3′) can promote non-homologous recombination to form rcAAVs [8]. However, our study shows that recombination can still occur at regions that lack Ad-ITRs, since the construct in our study does not contain an Ad-ITR at the 3′ end and the predominant forms found in Lot B lack Ad-ITR sequences at the 5′ junction. Thus, removal of the Ad-ITR from transgene cassettes appears to not be a viable strategy for eliminating rcAAVs. Importantly, our findings suggest that rcAAVs can form without sequence-specific recombination.

It was previously reported that separating the *rep* and *cap* ORFs onto distinct expression plasmids could eliminate the formation of rcAAVs [9]. However, these platforms have not been adopted widely for large-scale vector production schemes. Nevertheless, splitting the packaging elements onto separate expression vectors may not guarantee the elimination of rcAAVs, since recombination during the vector production process can occur quite frequently [15,16,28]. Therefore, SMRT sequencing of amplified rcAAVs found in preparations can help to understand how they form, which in turn will help to develop production platforms that are free of these potentially pathogenic contaminants. For example, one intriguing aspect of our investigation was the lack of full-length AAV-GOI sequences after just three passages of clarified lysates. This observation suggests that the AAV-GOI did not amplify along with rcAAVs or was not as efficiently replicated and packaged as well as the rcAAV species, despite the fact that the available genomes to be initially replicated are heavily dominated by the AAV-GOI vector. This finding may imply that poor replication of the AAV-GOI may be a dominant factor that promotes the formation and amplification of rcAAVs during production.

One clear limitation to our method is the bias in identifying unresolved truncated genomes. These species likely represent genomes undergoing replication or are unencapsidated double-stranded genomes. Genome ligation to the SMRTbell adaptors tends to favor species that have free double-stranded ends native to the strands or are end-processed during library construction [17]. Because of this, the distribution of reads does not faithfully capture the preparation population as revealed by gel electrophoresis or DNA fragment analyses by capillary electrophoresis. Nevertheless, the capacity for SMRT sequencing to cover full unit-length rcAAV genomes helps to validate their presence and can offer insights into their biology.

## Figures and Tables

**Table 1:** Alignment read counts.

Reference	Lot A	Lot B
Total reads	1,839,036	6,499,874
hg38 (human genome)	1,415,770 (76.98%)	5,393,393 (82.98%)
*rep*/*cap* plasmid	230,787 (12.55%)	217,501 (3.35%)
AAV-GOI	103,613 (5.63%)	31,127 (0.49%)

**Table 2:** Summary of 5′-recombination junctions found in rcAAV Lots A and B.

Lot A Forms	Sequence	Read Count (%) Total 3147
5′-end *rep*/*cap*	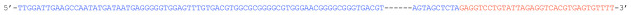	
A-5.1	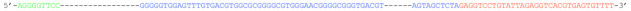	3073 (97.65%)
A-5.2	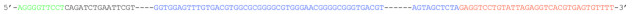	23 (0.73%)
A-5.3	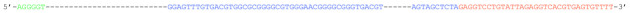	20 (0.64%)
A-5.4	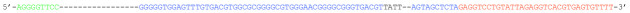	11 (0.35%)
A-5.5	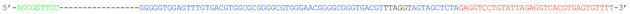	6 (0.19%)
A-5.6	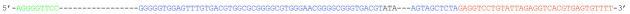	6 (0.19%)
A-5.7	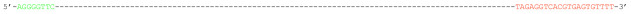	5 (0.16%)
**Lot B forms**		**Read count (%) Total 1019**
B-5.1	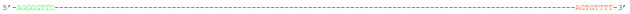	487 (47.79%)
B-5.2	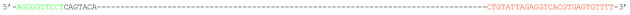	395 (38.76%)
B-5.3	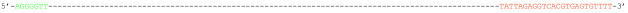	44 (4.32%)
B-5.4	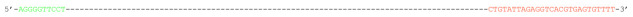	21 (2.06%)
B-5.5	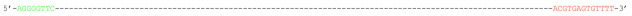	11 (1.08%)
B-5.6	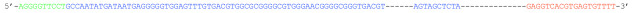	9 (0.88%)
B-5.7	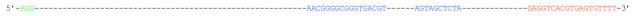	8 (0.79%)
B-5.8	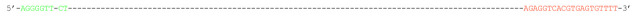	7 (0.69%)
B-5.9	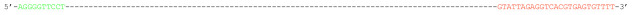	7 (0.69%)
B-5.10	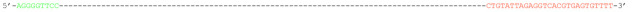	3 (0.29%)
B-5.11	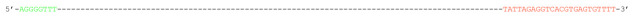	2 (0.20%)
5′-ITR (green) Partial Ad ITR (blue) AAV2 p5 promoter (red)		

**Table 3:** Summary of 3′-recombination junctions found in rcAAV Lots A and B.

Lot A Form	Sequence	Read Count (%) Total 3159
3′-end *rep*/*cap*	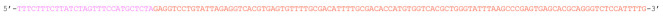	
A-3.1	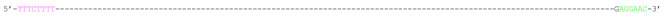	3,136 (99.27%)
**Lot B forms**		**Read count (%) Total 1058**
B-3.1	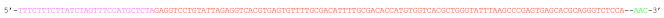	947 (89.51%)
B-3.2	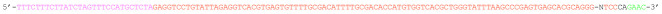	86 (8.13%)
B-3.3	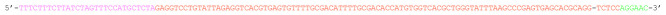	8 (0.76%)
AAV2 3′ end (magenta) AAV2 p5 partial insert (red) 3’-ITR (green)		

## Data Availability

The datasets generated and/or analyzed in the current study are available at the NCBI Sequence Read Archive (SRA) under the BioProject ID: PRJNA975558.
